# Genes Dysregulated to Different Extent or Oppositely in Estrogen Receptor-Positive and Estrogen Receptor-Negative Breast Cancers

**DOI:** 10.1371/journal.pone.0070017

**Published:** 2013-07-18

**Authors:** Xianxiao Zhou, Tongwei Shi, Bailiang Li, Yuannv Zhang, Xiaopei Shen, Hongdong Li, Guini Hong, Chunyang Liu, Zheng Guo

**Affiliations:** 1 Bioinformatics Centre and Key Laboratory for NeuroInformation of Ministry of Education, School of Life Science and Technology, University of Electronic Science and Technology of China, Chengdu, China; 2 College of Bioinformatics Science and Technology, Harbin Medical University, Harbin, China; 3 Genomics Research Center, Harbin Medical University, Harbin, China; 4 Department of Bioinformatics, School of Basic Medical Sciences, Fujian Medical University, Fuzhou, China; Karolinska Institutet, Sweden

## Abstract

**Background:**

Directly comparing gene expression profiles of estrogen receptor-positive (ER+) and estrogen receptor-negative (ER−) breast cancers cannot determine whether differentially expressed genes between these two subtypes result from dysregulated expression in ER+ cancer or ER− cancer versus normal controls, and thus would miss critical information for elucidating the transcriptomic difference between the two subtypes.

**Principal Findings:**

Using microarray datasets from TCGA, we classified the genes dysregulated in both ER+ and ER− cancers versus normal controls into two classes: (i) genes dysregulated in the same direction but to a different extent, and (ii) genes dysregulated to opposite directions, and then validated the two classes in RNA-sequencing datasets of independent cohorts. We showed that the genes dysregulated to a larger extent in ER+ cancers than in ER− cancers enriched in glycerophospholipid and polysaccharide metabolic processes, while the genes dysregulated to a larger extent in ER− cancers than in ER+ cancers enriched in cell proliferation. Phosphorylase kinase and enzymes of glycosylphosphatidylinositol (GPI) anchor biosynthesis were upregulated to a larger extent in ER+ cancers than in ER− cancers, whereas glycogen synthase and phospholipase A2 were downregulated to a larger extent in ER+ cancers than in ER− cancers. We also found that the genes oppositely dysregulated in the two subtypes significantly enriched with known cancer genes and tended to closely collaborate with the cancer genes. Furthermore, we showed the possibility that these oppositely dysregulated genes could contribute to carcinogenesis of ER+ and ER− cancers through rewiring different subpathways.

**Conclusions:**

GPI-anchor biosynthesis and glycogenolysis were elevated and hydrolysis of phospholipids was depleted to a larger extent in ER+ cancers than in ER− cancers. Our findings indicate that the genes oppositely dysregulated in the two subtypes are potential cancer genes which could contribute to carcinogenesis of both ER+ and ER− cancers through rewiring different subpathways.

## Background

Breast cancer is the most frequently diagnosed heterogeneous cancer among women in the world [1]. Two important subtypes are estrogen receptor-positive (ER+) and estrogen receptor-negative (ER−) breast cancers. They have different differentiation status and cell proliferation rates [2], [3], and behave distinctly in survival time [4] as well as in response to chemotherapy [5]–[7] and hormonal therapy [8]. To elucidate the molecular basis for the phenotypic differences between the two subtypes, many studies based on gene expression profiles have been performed to identify differentially expressed (DE) genes between the two subtypes [9]–[14]. These studies reveal that there are large-scale transcriptomic differences between ER+ and ER− breast cancers. For example, cell growth-related genes are predominately upregulated in ER+ cancer comparing to ER− cancer [13], whereas cell cycle related genes show predominantly higher expression in ER− cancer in comparison with ER+ cancer [14]. However, direct comparing the two subtypes cannot determine whether the DE genes result from dysregulated gene expression in ER+ cancers or ER− cancers in comparison to normal controls. In fact, a gene could be observed to be DE between the two subtypes in different situations: (1) the gene is dysregulated to a different extent of the same direction in the two subtypes, or (2) the gene is dysregulated in the opposite directions in the two subtypes, or (3) the gene is dysregulated in only one of the two subtypes. Gene expression differences from these situations might affect the two subtypes of breast cancer distinctly. Therefore, comparing genes dysregulated in ER+ cancers versus normal controls with genes dysregulated in ER− cancers versus normal controls could provide novel insights into the roles of the transcriptomic differences between the two subtypes.

In this study, we extracted DE genes of ER+ breast cancers (i.e., ER+ DE genes) versus normal controls and DE genes of ER− breast cancers (i.e., ER− DE genes) versus normal controls from microarray datasets. Because of the insufficient power of detecting DE genes, genes dysregulated in ER+ cancers only or in ER− cancers only could not be accurately defined. Thus, we focused on comparing genes dysregulated in both subtypes and classified these genes into two classes: class 1 DE genes and class 2 DE genes. Class 1 DE genes were dysregulated in the same direction and class 2 DE genes were dysregulated in the opposite directions. We showed these two classes of DE genes could be nonrandomly detected in independent RNA-sequencing (RNA-seq) datasets. Then, we classified the class 1 DE genes into two subclasses: genes dysregulated to a larger extent in ER+ cancers than in ER− cancers and genes dysregulated to a larger extent in ER− cancers than in ER+ cancers. We showed that the two subclasses of DE genes tended to enrich in distinct biological processes. We also proved that class 2 DE genes are potential cancer genes which could contribute to carcinogenesis of both ER+ and ER− cancers by rewiring different subpathways in the two subtypes.

## Materials and Methods

### Datasets and preprocessing

Microarray and RNA-seq data were downloaded from The Cancer Genome Atlas (TCGA) website (http://cancergenome.nih.gov/). Clinical characteristics of the samples analyzed in this study were summarized in Table 1. As it has been shown that the batch effects are “minimal” in the TCGA breast cancer datasets [15], a total of 519 primary female breast cancer samples with known ER status (401 ER+ and 118 ER−) and 63 normal controls were integrated into a microarray dataset from batches 47, 56, 61, 72, 74, 80, 85, 93, 96 and 103. Level 2 data of the platform Agilent 244 K Custom Gene Expression G4502A-07 (Agilent Technologies Inc., Santa Clara, CA, USA) were analyzed, in which log2 transformed and normalized expression values were provided. Probe sets with missing rates higher than 20% were deleted, and the remaining missing values were replaced by using the K nearest-neighbor imputation algorithm (k = 15) [16]. Probe sets were then annotated using the TCGA AgilentG4502A_07_3 annotation data file. Probe sets that did not match any known Gene ID or that matched multiple Gene IDs were deleted. For every sample, the expression values of the probe sets that were matched to the same Gene ID were averaged as the expression value of that Gene ID. We also analyzed the RNA-seq datasets from TCGA, which included a total of 787 primary female breast cancer samples (606 ER+ and 181 ER−) and 107 normal controls. These samples cover 564 of the 582 samples of the microarray dataset and another 330 samples (215 ER+ cancers, 66 ER− cancers and 49 normal controls) from recently available batches 109, 117, 120, 124, 136, 142, 147, 155, 167, 177, 202 and 216. Level 3 data of the platform Illumina HiSeq 2000 RNA Sequencing (Illumina Inc., San Diego, CA, USA) were analyzed, in which the RSEM (RNA-Seq by Expectation Maximization) [17], [18] calculated and normalized expression counts of each gene was provided. We then applied log_2_(x+1) transformation [19], [20] to the expression counts as they are often roughly log-normally distributed with an additional peak near zero [21].

**Table 1:** Clinical characteristics of the samples at diagnosis.

Characteristics	Microarray			RNA-seq		
	ER+	ER−	*p* *	ER+	ER−	*p* *
No. of patients	401	118		606	181	
Age(years)						
<55	151	64	1.31e-03	238	87	0.0350
≥55	250	54		368	94	
PR status						
Positive	334	7	1.35e-54	515	13	9.33e-85
Negative	67	111		91	167	
NA	–	–		–	1	
HER2 status						
Positive	61	21	0.506	89	34	0.169
Negative	332	95		484	136	
NA	8	2		33	11	
Tumor size						
≤2 cm	108	23	0.133	164	36	0.0783
>2 cm	290	91		423	134	
NA	3	4		19	11	
Node status						
Positive	202	52	0.219	307	76	0.0418
Negative	198	66		272	96	
NA	1	–		27	9	
Stage						
I/II	290	89	0.381	430	130	0.513
III/IV	98	24		144	38	
NA	13	5		32	13	
PAM50 subtype						
Luminal A	220	7	1.52e-67	218	6	1.21e-66
Luminal B	122	1		118	1	
HER2-enriched	33	25		31	25	
Basal-like	12	82		12	80	
Normal-like	6	2		5	2	
NA	8	1		222	67	

* *p* denotes results of significant test for the comparison between ER+ versus ER− breast cancer by chi-square test.

RNA-seq, RNA-sequencing; PR, Progesterone Receptor; NA, Not Available; HER2, Human Epidermal Growth Factor Receptor 2.

### Identification of DE genes

For both microarray and RNA-seq datasets, ER+ DE and ER− DE genes were identified between the normal samples and the two subtypes of breast cancer samples by using the SAM (Significance Analysis of Microarrays) (samr_2.0 R package, impute 1.32.0) [22], [23] with the false discovery rate (FDR) controlled at a given level by 10,000 permutation tests. The dysregulated direction of an ER+ DE or ER− DE gene was determined by the average expression difference, which was calculated by subtracting the average expression value of the normal samples from average of the ER+ or ER− cancer samples. A DE gene was defined as upregulated in cancers if expression difference was larger than zero. A DE gene was defined as downregulated in cancers if the expression difference was less than zero.

### Comparison of ER+ DE genes and ER− DE genes

If *N* DE genes were overlapped between *N1* ER+ DE genes and *N2* ER− DE genes and if *n* of the *N* overlapped genes were dysregulated in the same direction, then the *n* DE genes were defined as class 1 DE genes; the other *N-n* DE genes were defined as class 2 DE genes (i.e., genes dysregulated in the opposite directions in the two subtypes).

**Figure 1:**
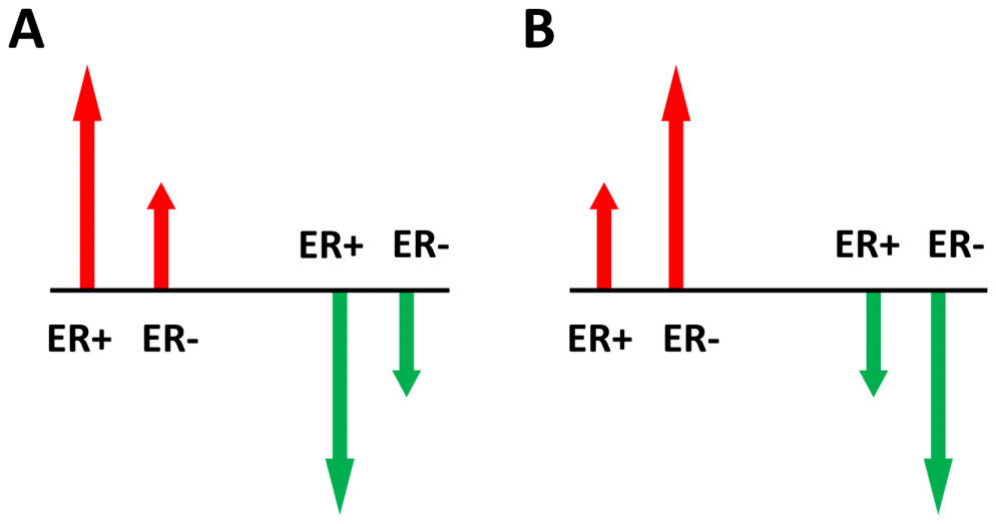
Schematic diagram of a gene dysregulated to a larger extent in ER+ (or ER−) cancer. Black line indicates average expression level of normal controls. Dysregulated directions are denoted in red arrow for upregulation and in green arrow for downregulation. The length of the arrow lines indicates dysregulated extent. (A) A gene dysregulated to a larger extent in ER+ cancer than in ER− cancer. (B) A gene dysregulated to a larger extent in ER− cancer than in ER+ cancer.

A class 1 DE gene was defined as dysregulated to a larger extent in ER+ cancers than in ER− cancers if it was upregulated (or downregulated) in both subtypes versus normal controls and if it was also upregulated (or downregulated) in ER+ cancers versus ER− cancers (Figure 1A), otherwise, it was defined as dysregulated to a larger extent in ER− cancers than in ER+ cancers (Figure 1B).

### Cancer genes and human protein-protein interaction (PPI) data

We downloaded 2104 cancer genes from the F-Census database [24] which is a collection of documented cancer genes from various data sources such as the CGC database [25], the AGCOH database [26], the TSGDB [27] and other data sources.

The human PPI data were downloaded from HPRD [28], IntAct [29], MIPS [30], MINT [31], DIP [32], BIND [33], KEGG (PPrel and ECrel) [34] and Reactome [35] protein pairs involved in a complex and neighboring reactions. The types of interaction relationships between proteins include physical interaction, transcriptional regulation and sequential catalysis. We pooled together the eight datasets and compiled an integrated interaction network of 235,390 distinct interactions involving 14,556 human proteins.

### Enrichment analysis

The Gene Ontology (GO) [36] gene annotation data and the GO vocabulary data were downloaded from the National Center for Biotechnology Information (NCBI) FTP Site (ftp://ftp.ncbi.nih.gov/gene/DATA) and the GO website (http://www.geneontology.org/GO.downloads.ontology.shtml) on March 25, 2011, respectively. Only the biological process sub-ontology was analyzed in this study. Biological processes enriched with a list of DE genes were identified by the GO-function algorithm [37], which is designed for handling the redundancy of GO terms. The statistical significance of a GO term is based on the hypergeometric distribution test with *p*-values corrected by the Benjamini-Yekutieli procedure [38]. The local redundancy was then treated when an ancestor term and its offspring term or terms were significantly enriched with DE genes. The ancestor term would be selected if there was evidence that its remaining genes were still related to breast cancer after removing the genes in its significant offspring term or terms; otherwise, only the offspring term or terms would be selected.

## Results

### Genes dysregulated to a different extent in ER+ and ER− breast cancers

Using the microarray dataset with a 1% FDR control, we identified 12,588 ER+ DE genes and 12,157 ER− DE genes in the ER+ and ER− cancer samples versus normal controls, respectively. The two lists of genes shared 9,734 genes, among which 93% (9,058 genes) were dysregulated in the same direction in the two subtypes (i.e., class 1 DE genes). We then validated the class 1 DE genes using an RNA-seq dataset with 330 samples of a different cohort, including 215 ER+, 66 ER− cancer samples and 49 normal controls. At the same FDR control level of 1%, we detected 6,006 class 1 DE genes in which 4,797 genes overlapped with the 9,058 class 1 DE genes of the microarray dataset. This was significantly more than expected by chance (*p*<2.2×10^−16^; hypergeometric test). For each of the overlapped genes, the dysregulated direction was identical in the two datasets for the ER+ and ER− cancers, respectively, which was unlikely to happen by chance if the dysregulated directions (up or down) of the shared DE genes were randomly assigned in the two datasets (*p*<2.2×10^−16^; binomial test). These results proved that the class 1 DE genes could be nonrandomly reproducibly detected across distinct datasets of different technologies. Because of the inefficient power of detecting DE genes, each dataset may capture only a fraction of the class 1 DE genes, but each of the gene lists were composed of mostly true class 1 DE genes [39], [40]. To increase statistical power, we identified ER+ and ER− DE genes in a larger RNA-seq dataset which includes the 330 samples and 564 of the 582 samples of the microarray dataset. With a 1% FDR control, we detected 7,948 class 1 DE genes of which 5,999 genes overlapped and showed the identical dysregulated directions with the 9,058 class 1 DE genes of the microarray dataset. Only the 5,999 class 1 DE genes confirmed in the RNA-seq dataset were used in the following analyses.

Given that the two subtypes were extensively different at the transcriptomic level, we then classified the 5999 class 1 DE genes into two subclasses: genes dysregulated to a larger extent in ER+ cancers than in ER− cancers and genes dysregulated to a larger extent in ER− cancers than in ER+ cancers (see Materials and Methods). In the microarray dataset, we found 2,151 class 1 DE genes that were dysregulated to a larger extent in the ER+ cancers than in the ER− cancers and 3,848 class 1 DE genes that were dysregulated to a larger extent in the ER− cancers than in the ER+ cancers. In the RNA-seq dataset, 1,746 (81%) of the 2,151 and 3,531 (92%) of the 3,848 genes were dysregulated to a larger extent in the ER+ and ER− cancer samples, respectively, which were highly unlikely to occur by chance if a gene dysregulated to a larger extent in ER+ or ER− cancers were randomly assigned for the two datasets (both *p*<2.2×10^−16^; binomial test). These results indicated that most of the class 1 DE genes were stably dysregulated to a larger extent in either ER+ or ER− cancers. By using the GO-function [37] with a 5% FDR control, we then found that the 1,746 genes were significantly enriched in 20 biological processes (Table 2). Besides transmembrane receptor protein tyrosine kinase signalling pathway and cell migration which had been found to be depended on oestrogen signalling in ER+ breast cancer [41], [42], these processes are mainly involved in glycerophospholipid and polysaccharide metabolic processes. Specifically, genes encoding enzymes of phosphorylase kinase family and glycosylphosphatidylinositol (GPI) anchor biosynthesis were upregulated to a larger extent in the ER+ cancers than in the ER− cancers, whereas genes encoding enzymes of glycogen synthase family and phospholipase A2 family were downregulated to a larger extent in the ER+ cancers than in the ER− cancers, suggesting that GPI-anchor biosynthesis and glycogenolysis were elevated and hydrolysis of phospholipids was depleted to a larger extent in the ER+ cancers than in the ER− cancers. In contrast, the 3,531 genes were significantly enriched in 22 biological processes (Table 3) which are mostly involved in cell proliferation and reflect the molecular basis of the higher proliferation rate of ER− cancers.

**Table 2:** The biological processes enriched with genes dysregulated to a larger extent in ER+ cancer.

Accession	GO Term	P-values	Q-values
GO:0044255	cellular lipid metabolic process	5.23E-08	5.03E-04
GO:0009888	tissue development	1.69E-07	7.63E-04
GO:0008610	lipid biosynthetic process	2.38E-07	7.63E-04
GO:0016477	cell migration	5.17E-07	1.02E-03
GO:0045017	glycerolipid biosynthetic process	5.28E-07	1.02E-03
GO:0016043	cellular component organization	1.01E-06	1.61E-03
GO:0009605	response to external stimulus	4.12E-06	2.84E-03
GO:0008654	phospholipid biosynthetic process	1.63E-05	8.26E-03
GO:0010033	response to organic substance	1.92E-05	9.02E-03
GO:0009719	response to endogenous stimulus	2.07E-05	9.06E-03
GO:0042476	odontogenesis	4.63E-05	1.75E-02
GO:0071845	cellular component disassembly at cellular level	4.64E-05	1.75E-02
GO:0007169	transmembrane receptor protein tyrosine kinase signaling pathway	4.74E-05	1.75E-02
GO:0009790	embryo development	5.03E-05	1.79E-02
GO:0016051	carbohydrate biosynthetic process	6.87E-05	2.28E-02
GO:0048583	regulation of response to stimulus	8.60E-05	2.70E-02
GO:0006650	glycerophospholipid metabolic process	8.70E-05	2.70E-02
GO:0048731	system development	1.76E-04	3.92E-02
GO:0044264	cellular polysaccharide metabolic process	1.83E-04	3.92E-02
GO:0048858	cell projection morphogenesis	2.01E-04	4.20E-02

**Table 3:** The biological processes enriched with genes dysregulated to a larger extent in ER- cancer.

Accession	GO Term	P-values	Q-values
GO:0051726	regulation of cell cycle	<2.2E-16	<7.7E-13
GO:0006259	DNA metabolic process	<2.2E-16	<7.7E-13
GO:0006974	response to DNA damage stimulus	<2.2E-16	<7.7E-13
GO:0006996	organelle organization	<2.2E-16	<7.7E-13
GO:0007049	cell cycle	<2.2E-16	<7.7E-13
GO:0051301	cell division	<2.2E-16	<7.7E-13
GO:0051329	interphase of mitotic cell cycle	<2.2E-16	<7.7E-13
GO:0007059	chromosome segregation	1.97E-14	6.59E-11
GO:0006950	response to stress	1.11E-12	3.16E-09
GO:0034622	cellular macromolecular complex assembly	4.73E-08	8.21E-05
GO:0016071	mRNA metabolic process	1.30E-07	2.21E-04
GO:0044267	cellular protein metabolic process	4.44E-07	7.06E-04
GO:0044419	interspecies interaction between organisms	6.81E-07	1.05E-03
GO:0051640	organelle localization	1.75E-06	2.48E-03
GO:0048522	positive regulation of cellular process	2.10E-06	2.90E-03
GO:0008283	cell proliferation	3.00E-06	4.02E-03
GO:0022613	ribonucleoprotein complex biogenesis	8.17E-06	1.01E-02
GO:0031145	anaphase-promoting complex-dependent proteasomal ubiquitin-dependent protein catabolic process	9.85E-06	1.17E-02
GO:0009411	response to UV	1.14E-05	1.33E-02
GO:0018209	peptidyl-serine modification	2.05E-05	2.24E-02
GO:0044265	cellular macromolecule catabolic process	2.41E-05	2.51E-02
GO:0043412	macromolecule modification	4.49E-05	4.34E-02

### Genes oppositely dysregulated in ER+ and ER− breast cancers

Of the genes dysregulated in both subtypes, 676 DE genes were oppositely dysregulated in the two subtypes of breast cancer from the microarray dataset (i.e., class 2 DE genes). We found that 163 of the 676 genes were also detected as class 2 DE genes in the RNA-seq dataset of 330 samples, which was significantly more than expected by random chance (*p*<2.2×10^−16^; hypergeometric test). In the larger RNA-seq dataset, we detected 720 class 2 DE genes in which 306 genes overlapped with the 676 class 2 DE genes of the microarray dataset (Table S1). In the following analyses, we focused only on the 306 class 2 DE genes. For each of these genes, we found that the dysregulated direction was identical in the two datasets for the ER+ and ER− cancers (Table S1), respectively, which was unlikely to occur by chance if the dysregulated directions of these genes were randomly assigned (*p*<2.2×10^−16^, binomial test). These results indicated that the class 2 DE genes could be nonrandomly reproducibly detected, which supported that each dataset may capture only a part of the class 2 DE genes, and each of the gene lists may comprise true class 2 DE genes [39], [40].

The opposite dysregulation of the class 2 DE genes implied that they might be cancer genes for the two subtypes of breast cancer, given that the expression of cancer genes tends to be differently dysregulated in the different subtypes of breast cancer [43], [44]. In fact, we found that 42 (14%) of the 306 genes were known cancer genes collected in the F-census database [24], which was significantly more than expected by chance (*p* = 0.03, hypergeometric test). In addition to the 42 known cancer genes, many other class 2 DE genes have been suggested to be proto-oncogenes or tumor suppressor genes in previous studies [45]–[49]. For example, knocking down *INPP4B* resulted in epithelial cell growth and overexpression of *INPP4B* led to reduced tumor growth [49], suggesting that *INPP4B* is a tumor suppressor gene. Furthermore, after removing the 42 cancer genes from the 306 class 2 DE genes, we found that the remaining 264 genes were significantly enriched in the direct interaction neighbors of the cancer genes collected in F-census (*p* = 0.04, hypergeometric test). This result implied that many of the remaining class 2 DE genes collaborated closely with the cancer genes and might function similarly as their interacted cancer genes during carcinogenesis. Thus, the class 2 DE genes are potential cancer genes for breast cancer.

**Figure 2:**
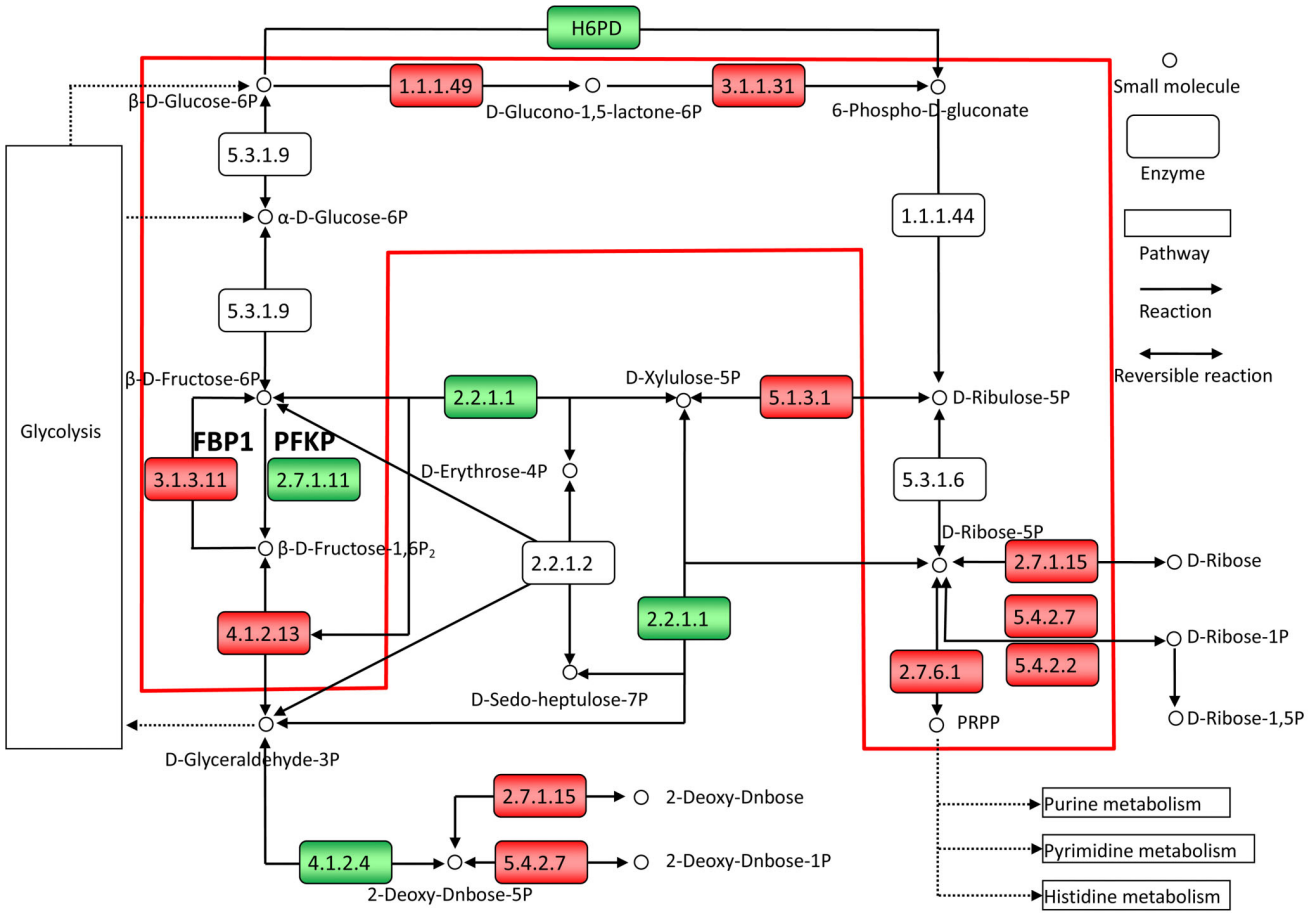
Downregulation of PFKP and upregulation of FBP1 contribute to ER+ breast cancers. ER+ DE genes in the pentose phosphate pathway are denoted in red for upregulation and in green for downregulation. The red frame indicates the elevated oxidative subpathway of the pentose phosphate pathway in ER+ cancers. The figure is created based on KEGG pathway hsa00030. Only a part of the pathway is shown for clarity.

**Figure 3:**
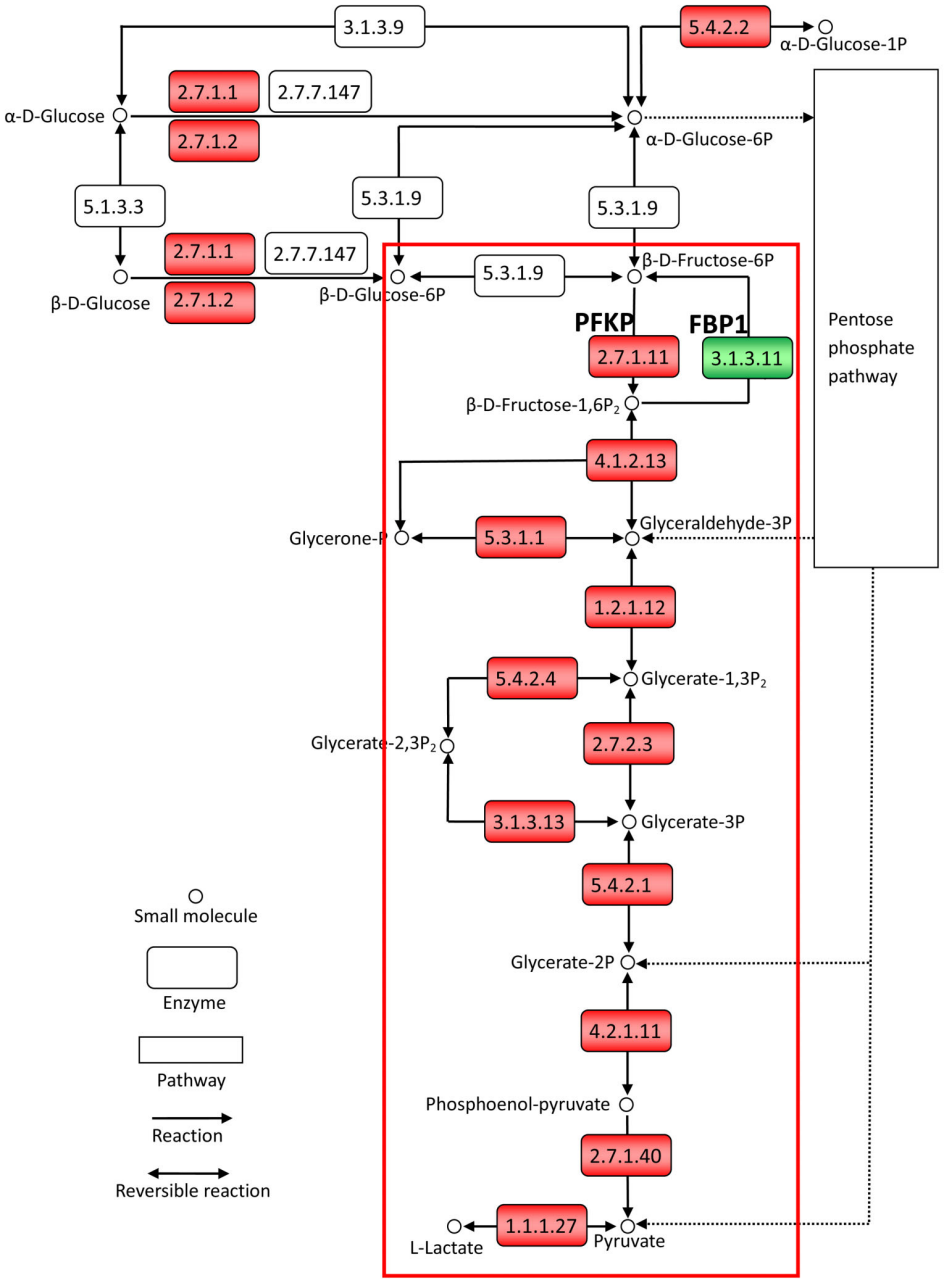
Upregulation of PFKP and downregulation of FBP1 contribute to ER− breast cancers. ER− DE genes in the glycolysis/gluconeogenesis pathway are denoted in red for upregulation and in green downregulation. The red frame indicates the elevated anaerobic glycolysis subpathway in ER− cancers. The figure is created based on KEGG pathway hsa00010. Only a part of the pathway is shown for clarity.

Genes oppositely dysregulated in the two subtypes may contribute to ER+ and ER− cancers through different subpathways. For example, *PFKP*, an estrogen signaling suppressive gene that encodes a rate-limiting enzyme of glycolysis [50], [51], was downregulated in ER+ cancers (Figure 2). This downregulation could induce the accumulation of fructose-6-phosphate [52]. The accumulated fructose-6-phosphate could then be converted into ribose-5-phosphate for synthesizing DNA and RNA since the key enzymes of the oxidative subpathway of the pentose phosphate pathway were upregulated in the ER+ cancers (Figure 2) [53]). This can contribute to the cell proliferation of ER+ cancers [54], [55]. By contrast, *PFKP* is upregulated in the ER− cancers, which could accelerate the rate-limiting step of the anaerobic glycolysis subpathway (Figure 3). The elevated activity of this step could provide abundant energy and substance which can support cancer cell proliferation [56], [57] since all of the downstream enzymes of the anaerobic glycolysis subpathway were upregulated in the ER− cancers (Figure 3). Therefore, the upregulation of *PFKP* also contributes to ER− cancers through the anaerobic glycolysis subpathway.

For another example, *FBP1*, an estrogen signaling responsive gene that encodes the enzyme catalyzing the reverse reaction of *PFKP* [58], [59], was upregulated in the ER+ cancers, which could elevate the activity of the oxidative branch of the pentose phosphate pathway and thereby contribute to cancers [60], [61] (Figure 2). By contrast, *FBP1* was downregulated in the ER− cancers, which could accelerate glucose metabolism and thereby contribute to ER− cancers through the anaerobic glycolysis subpathway [62], [63] (Figure 3). These two examples illustrate that an oppositely dysregulated gene could provide energy and substance for both ER+ and ER− cancers through different subpathways.

## Discussion

Although some studies have compared breast cancer subtypes with normal breast tissues using gene expression profiles [64]–[66], they mainly focused on the dysregulated genes in various subtypes and none of these studies compared directions of genes commonly dysregulated in different subtypes, especially in ER+ and ER− subtypes. In this study, we classified genes dysregulated in ER+ and ER− breast cancers into two classes and proved that the two classes of genes could be nonrandomly reproducibly detected from the microarray and the RNA-seq datasets of different cohorts. We showed that most of the genes dysregulated in the two subtypes were dysregulated in the same directions but to a different extent in the two subtypes (i.e., class 1 DE genes). We then classified the class 1 DE genes into two subclasses which enriched in distinct biological processes. Generally, glycerophospholipid and polysaccharide metabolic processes significantly enriched with the genes that were dysregulated to a larger extent in the ER+ cancers than the ER− cancers, while genes dysregulated to a larger extent in the ER− cancers were significantly enriched in biological processes involved in cell proliferation. Especially, phosphorylase kinase family and enzymes of GPI-anchor biosynthesis were upregulated to a larger extent in the ER+ cancers than in the ER− cancers, suggesting that these enzymes could be potential drug targets for breast cancer. For instance, inhibiting enzymes of phosphorylase kinase family might be an alternative way to suppress breast cancer growth. In fact, a recent study had demonstrated that targeting phosphorylase kinase could suppress angiogenesis in zebrafish [67]. Similarly, another study has showed that depletion of substrate of phosphorylase kinase, glycogen phosphorylase, causes glycogen accumulation, leading to tumor cell senescence and impaired tumor growth *in vivo* [68].

We also found 306 genes that were interestingly dysregulated in the opposite directions in the two subtypes (i.e., class 2 DE genes) for both microarray and RNA-seq datasets. The 306 class 2 DE genes significantly enriched with the known cancer genes and the rest genes that have not been documented in cancer gene databases tend to closely collaborate with the cancer genes, indicating that these genes are potential cancer genes. In addition, the genes upregulated in the ER+ cancers but downregulated in the ER− cancers included the previously found ER+/luminal expression signature genes [15], [69] and genes encoding MAPK signaling proteins and transcription factors. In contrast, the genes upregulated in the ER− cancers but downregulated in the ER+ cancers included genes encoding chemokines and cell adhesion molecules as well as apoptosis inhibitors. Many genes annotated in these functions had been demonstrated to be proto-oncogenes or tumor suppressor genes (TSGs) [45]–[49]. However, the confirmation of their proto-oncogene or TSG roles still needs further mutation experiments. In our recent study [70], we revealed that, in case-control experiments without considering genetic mutation (such as point mutation, insertion, deletion, copy number alteration) information, the expression levels of about one half of the "proto-oncogenes" are downregulated in cancer samples comparing to normal controls and about one half of the "TSGs" are upregulated in cancer samples. For a particular "cancer gene" (proto-oncogene or TSG), as genetic mutations usually occur in only a small proportion of cancer samples, its dysregulated direction detected in case-control experiments without genetic mutation information mainly reflects the expression change that occurs in samples with the wild-type counterpart [70]. Moreover, a gene can act as a proto-oncogene with activated mutations and it can also act as a TSG with inactivated mutations [70], [71]. Thus, we could not determine the class 2 DE genes played oncogene or TSG roles in the two subtypes as no genetic mutation information was available. Nevertheless, as the dysregulation of wild-type genes can still promote or support cancer cell growth, the opposite dysregulation of a class 2 DE gene could contribute to carcinogenesis of both ER+ and ER− cancers.

Because expression levels of the 306 oppositely dysregulated genes tended to correlate with ER status, their expression may potentially influence the sensitivity of ER+ cancers to adjuvant endocrine therapy [72], [73]. Thus, it is feasible to identify biomarkers based on these genes for predicting broad endocrine or specific agent resistance [74], [75]. Considering that predictive biomarkers for resistance to tamoxifen and/or aromatase inhibitors are essential to select the optimal adjuvant treatment for ER+ cancers and increase patient survival rates [76]–[78], it deserves our future researches.

As ER status is determined manually according to a certain percentage of ER+ cells using immunohistochemistry [15], some of the ER+ cancers contain ER− (basal) cells, and *vice versa*. A previous study showed that patients with 1% ER+ cells had significantly better survival compared with patients who had completely ER− cells, and survival also increased incrementally as the percentage of ER+ cells increased [79]. Recently, Iwamoto T *et al*. found that a minority of tumors with 1% to 9% ER+ cells show molecular features similar to those tumors with>10% ER+ cells, whereas most show ER− molecular characteristics [80]. These studies implied that expression levels of the class 1 and class 2 DE genes in ER+ cancer samples with low percentage ER+ cells would be similar to ER− cancers. To test this assumption, we compared lowest ER+ cell (1–19%) cancers with highest ER+ cell (90–99%) cancers and found that 97% (3,427) of the 3,531 genes dysregulated to a larger extent in the ER– cancers were also dysregulated to a larger extent in the lowest ER+ cell samples, and 99% (303) of the 306 class 2 DE genes were also oppositely dysregulated in the lowest and the highest ER+ cell samples. This analysis suggested that cell types and composition variation can also result in DE genes between sample groups. We then checked the component of cell types in tumor and normal samples and found that the average percentages of epithelial cell in cancer samples and normal samples were 84% and 74% [15], respectively. Potentially, more stromal cells were included in normal samples than cancer samples. Thus, a minority of ER+/ER− DE genes might be DE genes of epithelial cells and stromal cells. However, given that the two subtypes were compared to the same group of normal samples, this would not affect the extent and opposite expression differences between ER+ and ER− cancers.

Besides ER status, breast cancers are often classified into five intrinsic molecular subtypes [69], [81]–[85]. This taxonomy subdivides most of ER+ cancers into luminal A and luminal B subtypes while most ER− cancers belong to basal-like subtype. By contrast, the HER2-enriched subtype composes of almost equal number of ER+ and ER− cancers [15], while only a few breast cancers are normal breast-like subtype which may contain a disproportionately high content of normal epithelial and stromal cells [84], [86]. It has been found that luminal B cancers have a significantly worse prognosis than luminal A cancers [77], [87], [88] and have many similar molecular changes with the worst prognosis basal-like cancers, such as higher expression of proliferation-related genes [15], loss of the tumor suppressor RB1 [89] and higher kinase score [90]. These findings implied that expression levels of the class 1 and class 2 DE genes might be more closed to basal-like cancers (ER−) in luminal B cancers than in luminal A cancers. For the class 1 DE genes, among the 1,746 genes dysregulated to a less extent and 3,531 genes dysregulated to a larger extent in the ER− cancers than the ER+ cancers, 80% (1,401 genes) and 85% (3,012 genes) were also dysregulated to a less and a larger extent in the luminal B cancers than in the luminal A cancers, respectively. Additionally, among the 200 class 2 DE genes which were upregulated in the ER+ cancers but downregulated in the ER− cancers, 68% (136 genes) were upregulated to a less extent in the luminal B cancers than in the luminal A cancers. The expression similarities between the luminal B and the basal-like subtypes implied that luminal B cancers contain relatively less ER+/luminal cells and more ER−/basal cells than luminal A cancers. To verify this hypothesis, we divided the ER+ samples into two groups with high (≥ 50%) and low (<50%) percentages of ER+ cells and found that the luminal A and the luminal B subtypes significantly enriched with the high and the low ER+ cell groups (*p* = 0.0021, Fisher's exact test), respectively. This indicated that luminal B cancers tended to contain more ER−/basal cells than luminal A cancers, which could explain their worse survival compared with luminal A cancers, given that survival increases incrementally as the percentage of ER+ cells increasing [79].

One limitation of this study is that the statistical power of identifying class1 and class 2 DE genes could be low [91], [92]. Notably, it is known that, in the presence of small technical variations, the DE genes from two experiments tend to be inconsistent even if they are identified from two technically replicated microarray experiments using identical samples and mostly comprised true discoveries [39], [40]. This finding suggests that most of the two classes of DE genes identified from the two datasets might be true DE genes, although only a part of DE genes can be captured in each dataset due to the inefficient power. As a result, many of the two classes of DE genes detected in only the microarray dataset could actually be class 1 or class 2 DE genes in the larger RNA-seq dataset with increased power. To further verify this assumption, we roughly defined DE genes in the larger RNA-seq dataset using *t*-test with *p*<0.05 and identified another 2,836 “class 1” and 641 “class 2” DE genes. These genes overlapped 1,636 genes with the rest 3,059 class 1 and 199 genes with the rest 370 class 2 DE genes identified in the microarray but not in the RNA-seq dataset, respectively. Among the overlapped genes, 1,521 class 1 (92.97%) and 169 class 2 (84.92%) DE genes showed the same dysregulated directions in the microarray and the RNA-seq datasets for ER+ and ER− cancers, respectively, which were unlikely to happen by chance if the dysregulated directions of the rest DE genes were randomly assigned (both *p*<2.2×10^−16^, binomial test). Moreover, it had been proved that two DE gene lists are highly reproducible by considering expression-correlated or function-associated genes even though percentage of overlap between the two gene lists was extremely low [39], [40], [93]. Thus, we believe that most of the class 1 and class 2 DE genes identified in either microarray or RNA-seq dataset could be true.

## Supporting Information

Table S1 The 306 RNA-seq dataset validated oppositely dysregulated genes.
(XLSX)
